# Improving transitions in acute stroke patients discharged to home: the Michigan stroke transitions trial (MISTT) protocol

**DOI:** 10.1186/s12883-017-0895-1

**Published:** 2017-06-17

**Authors:** Mathew J. Reeves, Anne K. Hughes, Amanda T. Woodward, Paul P. Freddolino, Constantinos K. Coursaris, Sarah J. Swierenga, Lee H. Schwamm, Michele C. Fritz

**Affiliations:** 1 Department of Epidemiology and Biostatistics, College of Human Medicine, Michigan State University, B601 West Fee Hall, East Lansing, MI 48824 USA; 2 School of Social Work, College of Social Science, Michigan State University, East Lansing, MI USA; 3 Department of Media and Information, College of Communication Arts and Sciences, Michigan State University, East Lansing, MI USA; 4 Usability/Accessibility Research and Consulting, Michigan State University, East Lansing, MI USA; 5 Department of Neurology, Massachusetts General Hospital, Boston, MA USA

**Keywords:** Acute stroke, Case management, Transitional care, Caregiver, Randomized controlled trial, Social work

## Abstract

**Background:**

For some stroke patients and caregivers, navigating the transition between hospital discharge and returning home is associated with substantial psychosocial and health-related challenges. Currently, no evidence-based standard of care exists that addresses the concerns of stroke patients and caregivers during the transition period. Objectives of the Michigan Stroke Transitions Trial (MISTT) are to test the impact of a social worker home-based case management program, as well as an online information and support resource, on patient and caregiver outcomes after returning home.

**Methods:**

The Michigan Stroke Transitions Trial is a randomized, pragmatic, open (un-blinded), 3-group parallel designed superiority trial conducted in 3 Michigan hospitals. Eligible participants are adult acute stroke patients discharged home directly or within 4 weeks of being discharged to a rehabilitation facility. The patient’s primary caregiver is also invited to participate. Patients are randomized on the day they return home using a randomized block design. Consented patients discharged to a rehabilitation facility who do not go home within 4 weeks are dropped from the study.

The 2 study interventions begin within a week of returning home and conclude 3 months later. The 3-group design compares usual care to either a home-based social worker stroke case management (SWSCM) program, or a combination of the SWSCM program plus access to an online information and support resource (MISTT website). Outcomes data are collected at 7-days and 90-days by trained telephone interviewers. Primary patient outcomes include the PROMIS global 10 score (a generic Quality of Life scale), and the Patient Activation Measure (PAM). Caregiver outcomes include the Bakas Caregiving Outcomes Scale. Final analysis will be based on 214 randomized acute stroke patients. To accommodate subjects excluded due to prolonged rehabilitation stays, as well as those lost-to-follow-up, up to 315 patients will be consented.

**Discussion:**

The MISTT study will determine if a home-based case management program designed around the needs and preferences of stroke patients and caregivers, alone or in combination with a patient-centered online information and support resource can improve stroke survivor and caregiver outcomes 3 months after returning home.

**Trial registration:**

ClinicalTrials.gov: NCT02653170 (Protocol ID: 135457). Registered April 9, 2015.

## Background

Approximately 70% of the 1 million stroke patients discharged annually from U.S. hospitals return back to their homes. For many stroke patients and caregivers, navigating the transition between the hospital and home is associated with substantial emotional, social, and health-related challenges. Stroke is one of the most common reasons for needing daily in-home caregiving [1, 2] and many informal caregivers report high levels of stress and burden associated with the challenges of caring for stroke survivors [3–5]. These challenges are intensified by the abrupt nature of stroke, short hospital stays (typically <5 days), and the multiple care settings that patients use after leaving the hospital (rehabilitation, skilled nursing, home health care) [6–8]. As a consequence stroke patients commonly encounter high readmission rates, [9] slow or incomplete functional recovery, [10, 11] poor access to rehabilitation care, [12, 13] unmet educational and informational needs, [14] poor medication adherence, [15, 16] dissatisfaction with care, [17] and limited access to medical and community resources post-discharge [6, 8, 18]. The emphasis on minimizing hospital length of stay and the complex care system that follows hospital discharge means that many patients are unprepared for what follows and struggle to integrate back into the community [19–21]. The fact that transitional care for stroke patients in the US remains highly fragmented, poorly organized, and is rarely patient-centered, contributes to patient suffering and caregiver burden [8]. Furthermore, health outcomes important to stroke patients and their caregivers during the transitional period remain poorly characterized [6, 8].

One strategy for addressing transitional care challenges is to provide better education to patients. Stroke survivors and caregivers often report dissatisfaction with the quality and quantity of information provided to them, [18, 22, 23] and the desire to receive more high quality information about stroke [18, 23–26]. Many interventions have been designed to educate patients during hospitalization, [14, 27] yet it is recognized that this is not the optimal time to effectively absorb information [24]. Moreover, many of the in-home challenges patients and caregivers face cannot be fully appreciated or anticipated ahead of time, and thus cannot be addressed solely through a hospital-based approach. These facts make it clear that educational efforts need to occur during the post-discharge period. As an example, the disappointing results of a large trial of caregiver education and training in the U.K., [28] led to the recommendation that hospital-based education programs be integrated with home-based programs that can be provided over a longer time period [29].

Informational needs have been shown to exist up to 2 years after a stroke event, [25, 26] with the type of information and preferred format changing over time [23–25]. A recent systematic review concluded that educational programs customized to individual patient needs produce greater gains in knowledge and satisfaction than generic programs [14]. Due to the highly dynamic nature and longevity of the stroke transitional period, programs that deliver stroke education need to be flexible and easily adaptable; [24] one approach to doing this is to use web-based technologies. Interestingly, research on the use of computer-based stroke educational programs has been quite limited to date [30, 31].

Another strategy for addressing patient and caregiver transitional needs is to provide a case manager or transition coach - often a nurse or other para-professional [32, 33]. The transition-based intervention studies that have been effective in reducing readmissions and improving patient outcomes [33–35] have included few if any stroke patients, and did not collect patient-reported outcomes such as quality of life. Systematic reviews of intervention studies designed to improve transitional care for stroke patients have found limited effects of any given strategy, contain serious methodological limitations, and lack broad generalizability [27, 32] Thus there remains the need to design and test new patient-centered case management models to improve care transitions for stroke patients and caregivers.

To improve the transition experience of stroke patients and caregivers this trial is designed to address two critical needs: 1) access to a home-based case management program during the transition period, and 2) access to relevant information about stroke and available community services. The patient- and caregiver-centered home based case management program will be delivered by social workers. Social workers play a vital role in healthcare systems by determining the complex social, emotional, and medical needs of patients and connecting them to appropriate resources. The case management intervention will be tailored to the needs and preferences identified by patients and caregivers. We hypothesize that this personalized case management program will reduce unmet needs, improve quality of life, and decrease caregiver stress. To address the problem of accessing information about stroke and community resources during the transition period we will develop a website that will serve as a patient-centered online information and support resource. We hypothesize that this MISTT website will provide stroke patients and caregivers the information they need to improve their transition experience.

This study protocol follows the reporting standards developed by the SPIRIT (Standardized Protocol Items: Recommendations for Interventional Trials) statement; [36].

### Objectives

The central objective of the trial is to improve patient-centered outcomes following the transition home by testing two complementary interventions against usual care. The primary hypotheses are:

1. Compared to usual care, a home-based social worker stroke case management (SWSCM) program designed around the needs and preferences of stroke patients and caregivers will improve patient and caregiver outcomes 3 months after returning home.
2. Compared to a home-based social worker stroke case management (SWSCM) program only, access to the SWSCM program and the MISTT website - a patient-centered online information and support resource – will further improve patient and caregiver outcomes 3 months after returning home.

### Trial design

The Michigan Stroke Transitions Trial (MISTT) is a randomized, pragmatic, open (un-blinded), 3-group parallel designed superiority trial conducted in 3 Michigan hospitals. The 3-group parallel design will compare:

i) Usual care.
ii) A home-based social worker stroke case management (SWSCM) program.
iii) A home-based social worker stroke case management (SWSCM) program, plus access to the MISTT website which provides patient-centered information and support resources.

## Methods: Participants, interventions, data collection and outcomes

### Study setting

The study is being conducted in two Michigan cities located 65 miles apart: Lansing and Ann Arbor. In Lansing, stroke patients are enrolled at Sparrow Hospital, a comprehensive stroke center that serves as the sole tertiary referral center in the mid-Michigan region. Sparrow Hospital admits over 500 acute stroke cases per year. In the city of Ann Arbor, stroke patients are enrolled at two hospitals. The University of Michigan Teaching Hospital is a comprehensive stroke center that serves as a tertiary referral center for a large area of the state and admits over 250 acute stroke cases per year. Saint Joseph Mercy Hospital, Ann Arbor is a primary stroke center that serves as a tertiary referral center for the south-east region of the state and admits over 500 acute stroke cases per year. Combined, these 3 institutions admit approximately 1200 acute stroke patients a year, and represent both community based and academic medical centers.

### Target population - eligibility criteria

The target population for this pragmatic trial are all adult acute stroke patients discharged home either directly from the hospital or within 4 weeks of being discharged to a rehabilitation facility. Specific inclusion and exclusion criteria are described in Table 1. Eligible subjects need to have a final hospital discharge diagnosis of acute stroke (ischemic or hemorrhagic), present to the hospital with stroke-related deficits (confirmed by a National Institute of Health Stroke Severity [NIHSS] score of at least 1), be living at home prior to the stroke event, and be able to speak English. To identify patients who are more likely to have unmet needs after returning home, patients also need to have functional limitations at discharge, defined as a modified Rankin score [mRS] score of at least 1 at discharge or recommendations for post-discharge rehabilitative therapy.

**Table 1:** MISTT Study Patient and Caregiver Inclusion and Exclusion Criteria

Patient Inclusion Criteria:	
	i) A final confirmed hospital diagnosis of acute stroke (ischemic or hemorrhagic).
	ii) Patient living at home pre-stroke.
	iii) Presence of stroke-related deficits at admission (defined as a National Institute of Health Stroke Severity score of > = 1).
	iv) Presence of functional limitations at discharge (defined as a modified Rankin score [mRS] score of > = 1 or therapy ordered).
	v) Discharged directly home (includes patient’s residence or that of a family member), or discharged to a rehabilitation facility with the expectation of return to home within 4 weeks
Caregiver Inclusion Criteria:	
	i) Any person identified by the stroke patient as the primary caregiver (individual has primary responsibility for assisting with the patient’s care).
	ii) Age 18 or over.
Patient Exclusion Criteria:	
	i) Patients who live more than 50 miles from the hospital (for reasons related to the home visits).
	ii) Patients discharged to hospice care, nursing home for long term care, or long term care hospital (LTCH).
	iii) Patients who have clinically documented cognitive deficits or stroke-related impairments including aphasia sufficient to impact the consent process and for whom a proxy respondent is not available.
	iv) Patients who fail the 6-item Cognitive Screen for cognitive function (score < =4) and for whom a proxy respondent is not available.
	v) Patients enrolled in another acute stroke intervention trial that has a significant impact on the post-acute period (i.e., intensive data collection required of patient during follow-up).
	vi) Limited life expectancy (< 6 months) or significant medical comorbidity likely to impact completion of the study (e.g., severe mental illness, drug or alcohol abuse, metastatic cancer).
	i. Does not speak English.
Caregiver Exclusion Criteria:	
	i) Does not speak English.

### Subject enrollment - screening and consent

At each hospital site up to three trained study coordinators are responsible for conducting all screening, enrollment, and consent procedures using a 4 step process (Table 2). The study coordinators review the daily stroke census in combination with the admission notes to complete Step 1 (Pre-screening) which identifies all adult acute stroke admissions who live within 50 miles of the hospital. Step 2 (Screening) utilizes medical record data plus, if necessary, clarification with the clinical staff to confirm the patient meets eligibility criteria. At a suitable time, preferably with family members present, the coordinator approaches the patient to introduce the study and confirm eligibility. Step 3 (Final Confirmation) requires confirmation that the patient was living at home pre-stroke, that the patient speaks English, and that the patient is expecting to return home either directly or after going to a rehabilitation facility. If the patient and family express interest and eligibility is confirmed, the coordinator completes a 6-item cognitive screening (SIS-6,) [37] and proceeds with the consent process (Step 4). If the patient fails the cognitive screen (SIS-6 score < =4), or is unable to give consent because of aphasia, severe stroke-related deficits, or pre-existing dementia, a proxy respondent is sought. A proxy respondent may include any person who is authorized to make medical decisions on behalf of the patient; a proxy respondent may also be enrolled in the study as the primary caregiver. The consent process is typically completed while the patient is on the acute hospital ward, but for patients who are transferred to a rehabilitation setting consent may be completed there. For stroke patients who are discharged directly home before the coordinator has time to complete the consent process it may be completed by telephone.

**Table 2:** Steps for In-hospital Screening and Consent

Screening Steps	Data Source	Screening criteria	Data collected
Step 1 Pre- screening	Daily census and admission notes	Confirmed acute ischemic or Hemorrhagic stroke Adult. Lives <50 miles from hospital	Number screened
If pass Step 1, then			
Step 2 Screening	Chart & Attending Physician	NIHSS > = 1 at admission Lives at home mRS > = 1 at discharge or therapy ordered Discharge home or plan to go home within 4 weeks of going to rehab Life expectancy >6 months No other trial affecting follow-up No mention of cognitive deficits, significant stroke impairment or aphasia*	Screening log includes age, race, sex, stroke type, date of admission, and primary reason(s) I patient not eligible. Presence of clinical cognitive deficits, significant stroke related impairments, or aphasia.*
If pass Step 2, then			
Step 3. Final confirmation	Patient	Bedside confirmation of eligibility: live at home, plan to go home, speaks English. Complete Bedside 6-item cognitive screening.*	6-item cognitive screen score.* Document primary reason(s) patient not eligible
If pass Step3, then			
Step 4. Consent	Patient	Yes - agreed	Complete consent form
		No – declined	Document reason(s)
		No – not obtained ➔ study was introduced but no decision made about participation	Document reason(s)

*Proxy consent used if attending physician or chart mentions clinical cognitive deficits, significant stroke related impairments, or aphasia likely to impact the consent process (Step 2) or if patient has a 6-item cognitive screening score of <=4 (Step 3).

During the consent process, the coordinator asks the patient if there will be someone serving as their primary caregiver - defined as someone who will be providing regular assistance or help once they return home. If the patient identifies a primary caregiver and agrees to their involvement, the coordinator then contacts the caregiver to assess their eligibility (Table 1), determine their interest in participating, and obtain informed consent. The definition of a primary caregiver centers on the person who the patient believes, at the time of consent, will be most actively involved in their care; the person may be a spouse, family member, neighbor, or friend. The caregiver is not required to live with the patient. If the caregiver declines to participate in the study, the patient’s participation is not affected. Caregivers identified but not recruited prior to the patient’s discharge home will be contacted for consent up to 10 days post-discharge either by phone or in person (if the patient is randomized to receive case management). If a primary caregiver is not identified until after the patient is discharged home recruitment is not pursued.

### Interventions

The 3-group parallel design compares usual care to either a home-based social worker stroke case management (SWSCM) program, or a combination of the SWSCM program plus access to the patient-centered online information and support resource (MISTT Website). The interventions begin shortly after the patient is discharged to home and end no later than 3 months post-discharge. The format and content of both interventions (SWSCM program and the study website) were guided by input from focus groups conducted with stroke patients and caregivers during the pre-trial development phase, as well as the experience gained during a pilot test phase.

Group 1: Usual care: Patients in this group receive the hospitals’ usual care approach as documented in the discharge instructions. For the 3 hospitals this includes the typical combination of post-acute care services for stroke patients, including education materials, appointments to see out-patient medical providers (e.g., primary care, neurology, rehabilitation), and referrals to other community-based providers (e.g., home care). Although some hospitals utilize hospital-based programs designed to reduce hospital readmissions, none of these programs specifically target acute stroke patients. To promote retention in the study, patients assigned to the usual care group receive mailings of stroke and health related brochures. The first mailing is sent within a week of returning home, and then two additional mailings are sent after 4 and 8 weeks.

Group 2: SWSCM: Four Masters-level social workers were trained to serve as Social Worker Stroke Case Managers (SWSCM) for this intervention. The broad goals of the intervention are to provide case management services for between 60 and 90-days following the return home. Typically this will involve at least 2 home visits–the first visit occurring within a week of returning home, and the second scheduled about 30-days later. The SWSCMs also follow up weekly with each patient by phone. The specific case management services provided are dictated by the patient and caregiver needs and preferences so the exact number of home visits and follow-up telephone calls are determined on a case by case basis. The intent is to complete services by 60-days, however the program is continued for up to 90 days if ongoing achievable goals exist. During the intervention period, the SWSCM conducts the following activities:

1. Within the first week post-discharge, undertake a home visit to conduct a *biopsychosocial assessment* [38] to identify patient and/or caregiver goals and unmet needs identified as most important to them. If a patient declines to have a home visit, this assessment can be conducted by phone.
2. Develop a patient-centered *service plan* with specific actions that address the goals and unmet needs. This plan is used throughout the intervention period, and is revised as new unmet needs and goals are identified or prior needs and goals are resolved.
3. Review the patients’ discharge paperwork and incorporate activities into the service plan as necessary.
4. Ensure that the patient is seen by a primary care physician after discharge (preferably within 7-days of discharge). Set up appointment if not already scheduled. Assist with setting up appointments with other medical providers as needed.
5. Make referrals to community agencies including signing up patient for financial assistance programs (e.g. medical, food, housing) as necessary.
6. Promote medication adherence through education involving use of medication lists, tool kits, pill organizers, and other aids.
7. Facilitate patient and caregiver engagement and activation through support and education related to their unmet needs and emphasizing the importance of stroke prevention and recovery.
8. Facilitate access to social and community services as necessary (determined by their unmet needs).
9. Maintain an interaction log to document all interactions, activities and referrals.
10. Complete a final close out case summary between 60 and 90-days.

The *biopsychosocial assessment* will identify the current needs of the patient and caregiver [38]. These needs will range in severity and can include: [39–42]

1. Out-patient Medical Services e.g. appointments with primary care physician, specialists, access to rehabilitation care, access to home health care, transportation, access and adherence to medications.
2. Psychological e.g. stress, depression, anxiety, negotiating self-understanding, coping.
3. Social e.g. role participation and shifts, isolation, need for support, communication with caregivers.
4. Physical e.g. home safety, functional abilities, fall risk, self-care, durable medical supplies.
5. Information/Services e.g. transportation, financial, medical insurance, meals, cleaning services, healthy lifestyle.

After the service plan is developed and patient/caregiver goals and unmet needs identified, the SWSCM works to identify specific resources, suggest solutions to specific problems, and identify and resolve specific barriers. During weekly follow-up phone calls the case managers monitor progress, provide further information and resources, and help coordinate services as necessary. The SWSCMs encourage the patient to actively pursue services and resources on their own but will, depending on the level of function, assist them directly if needed.

At about 60 days after returning home, if the SWSCM, patient and caregiver agree that no further help is required, or that an adequate plan has been initiated for needs requiring longer-term management, the case is closed out and a final close out case summary is completed. If necessary, case management can be continued for up to 90 days. The close out report summarizes the progress made during the intervention period, documents any remaining unmet needs including follow up recommendations, and describes the overall level of compliance and engagement the patient and caregiver had with the SWSCM. Compliance with specific SWSCM recommended tasks and activities are documented in the service plan, case notes, and interaction log maintained by SWSCM. The level of involvement may vary based on the number of identified unmet needs and the patient’s and/or caregiver’s preferences for interactions. If a patient is admitted to a hospital, rehabilitation facility, or another setting where it is not possible to deliver the intervention (e.g., nursing home) then the intervention is temporarily suspended until the patient returns home. If the 90-day study deadline is reached while the subject is still away from home then the intervention ends.

Group 3: SWSCM plus MISTT Website (Online Information and Support Resource) – In addition to the SWSCM activities described above, this group will also receive access to and training in the use of the MISTT website - a purpose-built, online, patient-centered information and support resource. The website was designed and developed by the MISTT research team, and underwent usability tested with a group of representative stroke survivors and their caregivers. The website contains the following components:

1. Stroke related information – Provides links to stroke education materials, resources and guidelines developed by the National Stroke Association, [43] the American Stroke Association, [44] and other organizations. Resources to promote patient activation and self-management related to stroke prevention and recovery are also included.
2. Medication information - Provides access to materials to promote medication adherence including patient education materials for stroke-related medications, drug-to-drug interaction, and tracking and reminder tools including templates, online diaries, and a smartphone app.
3. Provider contact list - To promote communication between the patient, caregiver and their care team, the website organizes contact information for each care team member that the patient identifies. This list is set up with the assistance of the SWSCM.
4. Hospital patient portal - The website will encourage patients to access their medical record as well as other hospital resources through the hospital’s patient portal (EMR). Patients and caregivers will be encouraged to register and shown how to use their portal.
5. Access to community resources– - Provides links to the *Michigan 2–1-1* web site [45] which provides access to searchable information on local social services and resources useful for both patients and caregivers. Patients and caregivers can also access services by calling a 2–1-1 operator.
6. Stroke Support Groups– Provides information on local face-to-face support groups as well as online stroke support groups (discussion forums and chat-rooms) that include other stroke survivors and caregivers, for example, online support groups supported by the National Stroke Association [46].
7. Caregiver resources -- This section includes specific information and resources designed to help caregivers with their tasks as well as access to caregiver networks and support groups.

The SWSCM typically introduces the website to the patient and caregiver at the first home visit. This includes an assessment of the patient’s and caregiver’s readiness to use the website, which is followed by a demonstration on how to access the website using their preferred technology platform (desk top, laptop, tablet, smartphone). Introductory videos that explain the purpose, organization and specific content of the website are included on the website. Access to the website requires the patient or caregiver to log-in with an e-mail address and password; if the patient and/or caregiver does not have an existing e-mail, the SWSCM will set one up. If there is no in-home access to the internet then the project will supply a tablet computer with internet access for the duration of the intervention. During follow-up calls and home visits the SWSCM provides further encouragement to access and use the website. Adherence with the MISTT study website component is monitored remotely by recording the frequency that patients and caregivers access specific pages of the website. This data is based on their unique log-in and collected using Google Analytics.^1^

### Intervention monitoring, adherence and withdrawals

As noted above, adherence to the home-based case management intervention is monitored and documented by the SWSCMs in their case notes and interaction log which are recorded in the REDCap study database.^2^ The interaction log documents the method, duration, target, and primary task of each interaction. All SWSCM documentation is reviewed by the social work team leader (AH) to assess accuracy, completeness, and consistency with the patient-centered case management intervention. All SWSCMs are trained on the requirements of the intervention and expected documentation; training updates are provided throughout the trial to prevent intervention drift. SWSCMs meet bi-weekly with the social work team leader to discuss problem cases and solutions. Because this is a patient-centered intervention the SWSCM maintains a flexible approach to the content, frequency and nature of the interactions depending on the patient’s and caregiver’s needs and expressed wishes. If a patient and/or caregiver is unable to meet with the SWSCM in person either because of inconvenience (e.g., conflicts due to work, family or therapy visits), or because they have temporarily moved out of the service region [>50 miles]) then an attempt is made to continue the intervention via telephone. As described earlier the intervention is temporarily suspended when a subject is readmitted to the hospital, or admitted to another type of facility during the intervention period. The intervention will re-start if the patient returns home within the original 90-day intervention time window (calculated from the original discharge to home date).

Patients who refuse to cooperate with the SWSCM for a period of at least 4 weeks, or who request to no longer receive the intervention are closed out at that time. These subjects are referred to as a *voluntary withdrawal from intervention* but are still eligible to receive a final 90-day outcome call (unless they specifically decline this option). The intervention is permanently discontinued if the subject dies, is admitted to hospice, permanently moves out of the service area (and is unwilling/unable to participate via phone), or requests to be withdrawn from the study completely. These subjects are referred to as an *administrative withdrawal from the study* and do not receive the final 90-day outcome call.

### Concomitant interventions

This trial is conducted within the milieu of usual and customary care provided to stroke patients in the post-acute setting; the MISTT interventions are an adjunct service and do not replace any standard-of-care services. Thus, stroke patients continue with their post-discharge care plan set by their medical care team as outlined in the discharge plan and modified by their primary care physician or other providers. Patients are therefore expected to continue to access out-patient rehabilitation, home health care services, and specialty office visits as prescribed by their care team.

### Outcomes data

Patient outcome measures were selected after an extensive review of available instruments relevant to functional recovery, disability, handicap, and quality of life of stroke patients and caregivers. We relied heavily on patient-reported functional and QOL measures developed by the PROMIS (Patient-Reported Outcomes Measurement Information System) and NeuroQOL programs [47–50]. The PROMIS and NeuroQOL programs are National Institutes of Health (NIH) initiatives to develop and validate patient-reported outcome measures that can be applied to a wide variety of diseases, conditions and settings [47, 50]. The HealthMeasures website provides access to a comprehensive set of items banks, short forms, and computerized adaptive tests (CATs) that measure various aspects of physical, mental, and social health [47].

The final set of patient measures were classified into primary outcomes, secondary outcomes, mediating (intermediate) outcomes, and potential effect modifier variables (Table 3). There are two primary patient outcome measures: the PROMIS global 10 score, a generic quality of life scale, [47] and change in the 13-item Patient Activation Measure [PAM], a measure of patient self-efficacy [51]. Secondary patient outcome measures include depression symptoms (PHQ-9), NeuroQOL anxiety scale, 90-day hospital readmissions, 90-day stroke recurrence, and 90-day home time [52]. Mediating (intermediate) measures include PROMIS self-efficacy scales (emotions, daily activities, social engagement, medication management), PROMIS support scales (informational, emotional, instrumental), [47] and utilization and barriers to computer use (Table 3).

**Table 3:** Patient Outcome Measures

	When Measured			
PRIMARY OUTCOMES	Baseline	7 day	90-day	No. questions
PROMIS Global-10 QOL		X	X	10
Patient Activation Measure [PAM]		X	X	13
SECONDARY OUTCOMES				
PHQ-9 depression		X	X	9
NeuroQOL anxiety		X	X	CAT*
Hospital/ED visits – self report		X	X	1
Stroke recurrence – self report			X	1
90d home time			X	1
MEDIATING (INTERMEDIATE) OUTCOMES				
PROMIS self-efficacy – manage social interaction		X	X	4
PROMIS self-efficacy – manage medications		X	X	5
PROMIS self-efficacy – manage emotions		X	X	CAT*
PROMIS self-efficacy – manage daily activities		X	X	CAT*
PROMIS informational support		X	X	5
PROMIS instrumental (practical) support		X	X	5
PROMIS emotional support		X	X	5
Dyad relationship scale			X	10
Unmet needs self-report			X	1
Stroke-specific technology use			X	8
EFFECT MODIFIER VARIABLES				
Demographics (age, sex, race, insurance, housing, living alone)	X			6
Demographics (marital status, education, home health care)		X		3
Pre-stroke function and ambulation	X			2
Past medical history/comorbidities/risk factors	X			11
Stroke type (ischemic, hemorrhagic)	X			1
Admission stroke severity (NIHSS, level of consciousness, symptom duration)	X			3
Clinical treatments (thrombolytics, endovascular or other surgical intervention)	X			3
Discharge instructions, rehab and home health prescriptions	X			3
Discharge Modified rankin and FIM score	X			2
Pre-stroke function and ambulation		X		2
Modified rankin (current function)		X	X	3
ADL and IADL (current)		X	X	12
Care Transitions Measure CTM-3		X		3
SIS 6-item cognitive screener	X	X	X	6
Pre-stroke depression diagnosis		X		2
NeuroQOL communication		X	X	5
NeuroQOL emotional & behavioral dyscontrol		X	X	CAT*
Pre-stroke technology utilization		X		10
Computer anxiety		X		2
Computer self-efficacy		X		4
Satisfaction with Interventions			X	2

*CAT = Computer Adaptive Testing. Number of questions dependent on individual response. Average is about 6.

Caregiver outcome measures were similarly classified (Table 4). There are two primary caregiver outcome measures: the Bakas Caregiving Outcomes Scale (BCOS), [53] and depression symptoms (PHQ-9). Secondary caregiver outcome measures include: unhealthy days, and PROMIS support scales (informational, emotional) [47]. Mediating (intermediate) measures include preparedness for caregiving scale, [54] and the Oberst Caregiver Burden Scale (OCBS) [55].

**Table 4:** Caregiver Outcome Measures

	When Measured		
PRIMARY OUTCOMES	7 day	90-day	No. questions
Bakas Caregiver Outcomes Scale	X	X	16
Depression (PHQ-9)	X	X	9
SECONDARY OUTCOMES			
Unhealthy days		X	2
PROMIS informational support	X	X	5
PROMIS emotional support	X	X	5
MEDIATING (INTERMEDIATE) OUTCOMES			
Preparedness for Caregiving Scale	X	X	9
Oberst Caregiving Burden Scale	X	X	14
Dyad relationship scale		X	11
Unmet needs – self-report		X	3
Stroke-related technology use		X	8
EFFECT MODIFIER VARIABLES			
Demographics (age, sex, race; marital status, education, employment, household income)	X		7
Change in employment, household income		X	3
Relationship with patient	X		3
SIS Cognitive Screen	X	X	6
Pre-stroke depression diagnosis	X		2
PROMIS Global 10 QOL (proxy report of patient)	X	X	10
Pre-stroke technology utilization	X		10
Computer anxiety	X		2
Computer self-efficacy	X		4
Satisfaction with Interventions		X	2

### Participant timeline

A summary of the expected timeline for participant involvement is shown in the Table 5. Enrollment is expected to occur during the acute hospital stay (or shortly thereafter) for both patients and caregivers. Randomization occurs on the day the subject returns home (Study Day 1). Interventions are scheduled to begin within one week (Study Day 7) and conclude after 3 months (Study Day 90). Baseline clinical data are collected during the in-hospital stay and outcomes data are collected in 2 telephone interviews scheduled around Study Day 7 and 90. The 7 day interview is designed to collect baseline data on function and quality of life soon after the patient and caregiver have returned home. Ninety days was chosen for the final outcome assessment as this is a common time point used in many clinical stroke trials and is regarded as a benchmark of early recovery. Ninety days is also a logical choice given that the case management program is designed to end after 60-days (although it can continue up to 90-days).

**Table 5:** Summary of typical participant timeline including enrollment, interventions and assessments

Study Time Period		Study Day 1	Study Day 7 (first week after returning home)		Study Day 8 to Day 90 (second week to week 12)			
	Acute Hospital Stay	Discharge home (Day 1)	First SWSCM Home visit (Day 7)	Baseline phone call (Day 7)*	Second SWSCM Home visit (Day 30)	SWSCM Follow-up calls and visits (ends Day 60–90)	Final Outcome phone call (Day 90)*	Goggle Analytics (7–90 days)
ENROLLMENT								
Screening (Steps 1–3)	X							
Patient Consent (Step 4)	X							
Caregiver Consent	X		X					
Randomization		X						
INTERVENTIONS								
SWSCM Intervention								
Biopsychosocial assessment			X					
Establish service plan			X					
Update service plan					X	X		
Maintain interaction log					X	X		
Close out assessment						X		
MISTT website								
Orientation			X					
Reminders					X	X		
ASSESSMENTS								
Baseline clinical data collection	X	X						
Outcomes Data Assessment				X			X	
MISTT Website Utilization								X

*Timing of phone calls vary between day 5 and 21 (for baseline) and between day 83 and 111 (for final).

### Sample size

Sample size estimates were calculated based on the two primary patient outcome measures: the PROMIS global 10, [47] and the PAM [51]. All estimates were generated in SAS [56] using a one-way ANOVA model with power 80% and alpha 0.05. Because there is little data on the use of the PROMIS global 10 measure in stroke populations to inform calculations (i.e., data on responsiveness or minimal important difference), we generated estimates based on the stroke-specific QOL (SS-QOL) measure [57] with anticipated differences in the summary SS-QOL score obtained from a previous stroke study [58]. Using a hypothesized largest between group difference of 1.0 in the summary score with a standard deviation of 1.9 (equivalent to an effect size of 0.52) resulted in a sample size estimate of 214 total subjects. Varying the standard deviation between 1.6 and 2.5 (equivalent to effect sizes of 0.62 and 0.40), resulted in total sample sizes of 154 and 366, respectively. A widely used rule of thumb is to use an effect size of 0.5 as a benchmark for clinically meaningful change for many types of QOL measures [59, 60]. Thus, we believe the sample size calculation of 214 (based on an effect size of 0.52) should be sufficient for the PROMIS 10 measure.

Validation work has shown that an average PAM score is 62 with a standard deviation of 13, [51] and that changes of 5 are clinically meaningful [61]. Using a hypothesized largest between group difference of 10 with a standard deviation of 13 (equivalent to an effect size of 0.77) resulted in a sample size estimate of 102 total subjects. Varying the standard deviation between 10 and 15 (equivalent to effect sizes of 1.0 and 0.67), resulted in sample sizes of 64 and 136, respectively. Thus the sample size estimate of 214 calculated for the PROMIS global 10 outcome will be more than sufficient for the PAM outcome.

### Recruitment

Although our target final sample size is 214 subjects available for analysis, we anticipate that about 15% of subjects will drop out after randomization either because they are withdrawn from the study (due to death, hospice, moved away, or declined) or because they are lost-to-follow-up at 90-days. Thus the target number of randomized patients was increased to 252. We also estimate that up to 20% of consented patients will be dropped from the study prior to randomization because they remain in a rehabilitation facility for longer than 4 weeks (modified intention-to-treat design). To accommodate we increased the target number of patients to be consented by 20% to 315 (105 per site). Using an 18-month recruitment period this translates to 6 cases per month per hospital. To monitor recruitment, monthly audits of each sites’ enrollment numbers are generated and discussed during regular group conference calls. Site visits are conducted on an as-needed basis to help problem solve recruitment issues.

## Methods: Assignment of interventions (randomization)

### Randomization

The target number of patients to be randomized is 252. Patients are randomized on the day they return home. To ensure that allocation to the 3 treatment groups is balanced within each hospital, randomization is stratified by hospital. Sequence generation is performed centrally using a computer generated randomized block design (with block size of 6 or 9) housed within a secure REDCap data management system. Allocation concealment is ensured by the fact that randomization is not performed until the project manager (MF) receives confirmation that the patient has returned home. The project manager is responsible for all aspects of implementation and communicates the results for each randomization to the care team by e-mail.

### Blinding

This is an open trial – it is not possible to mask study participants or any of the study staff to the intervention group assigned to a given patient. It was originally intended that the telephone interviewers collecting outcomes data were to be blinded to group assignment, however, during the pre-trial phase this proved to be impractical. The potential for interviewer bias is ameliorated by the fact that the telephone-based data collection instrument contains validated patient reported outcome measures with standardized response options which do not require interviewer interpretation or observation.

## Methods: Data collection, management and analysis

### Data collection methods

Relevant patient- and caregiver- reported outcomes data organized in terms of primary outcomes, secondary outcomes, mediating (intermediate) outcomes, and potential effect modifier variables are summarized in Tables 3 and 4, respectively. These data are collected through a combination of medical record abstraction and telephone interviews.

### Medical record abstraction

After a patient is discharged from the hospital, baseline clinical data are abstracted from the medical record by the trained hospital coordinators. The data includes demographics (age, race/ethnicity, sex), insurance status, pre-stroke address and living arrangements (living alone), pre-stroke function and ambulatory status, past medical history, comorbidities, risk factors, stroke type, stroke severity and symptom duration at admission, thrombolytic and endovascular treatments, other neurosurgical interventions, discharge FIM score, discharge mRS, and discharge summary including therapy orders. (Table 3) Additional patient demographic data on marital status and education are collected at the first telephone interview post discharge. No clinical or laboratory assessments are collected for the purposes of this study. Demographic data collected from caregivers includes age, race/ethnicity, sex, marital status, level of education, income, employment status, and living arrangements. (Table 4) These data are collected during the consent process or at the first telephone interview.

### Telephone interview data

Patient- and caregiver- reported outcomes data are collected by trained telephone interviewers at two time points: 7 days (range 5–21 days), and 90-days (range 83–111 days) after returning home. Separate survey instruments for patients (Table 3) and caregivers (Table 4) include a comprehensive set of clinical and subject-reported functional and QOL measures, many selected from the PROMIS and NeuroQOL question banks [47–50]. The full interviews take approximately 30 min (range 20–60) to complete. Proxy versions of the 2 patient interviews were also developed and both the 7-day and 90-day caregiver instruments also include a proxy assessment of the patient’s current quality of life (based on the PROMIS Global 10 measure).

As described earlier, only subjects who are administratively withdrawn from the study (i.e., due to death, admission to hospice, permanent move from service area, or request to stop all study participation) do not receive the final 90-day telephone interview. Subjects who did not complete all of the intervention as planned (i.e., voluntary withdrawals) are still eligible to complete the final interview. Completion of the final 90-day interview is promoted by sending a reminder letter just prior to the due date, and giving a $25 gift card for completing the call. We also developed abbreviated versions of each instrument for subjects who are physically unable or refuse to complete the full interview.

### Data management

All study data are entered into a secure REDCap database, [62] which includes web based data entry platforms for hospital staff to enter medical record information, and SWSCMs to document case management activities (assessments, case notes, interaction log). The system also allows direct data entry during the telephone interviews. All study personnel use secure passwords to access the database. Control of read and write privileges allow study coordinators and the SWSCMs to only view data for patients from their study site; similar read and write privileges control access to all protected health information (PHI). To ensure data quality, data fields are pre-programmed to restrict out of range or implausible data.

### Statistical methods

Descriptive statistics including frequencies and contingency tables for categorical data, and means, medians, standard deviations and ranges for continuous data will be generated for all variables. The adequacy of randomization will be examined by testing for between group differences in demographic and clinical factors (e.g., age, stroke type, NIHSS). If outcomes data are missing for more than 10% of randomized cases then we will explore the use of multiple imputation methods using the PCORI Methodology Standards as guidance [63].

The final sample size target for analysis of the primary trial outcomes is 214 subjects. Consented subjects who do not go home within a month because they remain in a rehabilitation facility will be dropped from the study; however, these subjects do not impact the validity of the study as they do not undergo randomization prior to their exclusion. To accommodate these pre-randomization losses, as well the post-randomization losses that occur when cases are administratively withdrawn from the study or are lost-to follow-up, the target number of subjects to be consented is increased to 315. Among the cases available for analysis an intention-to-treat analysis, where all subjects are analyzed according to the group to which they were randomized, will be followed.

The primary analysis of this 3-group parallel designed trial will rely on a generalized linear model framework [64] (one-way ANOVA for continuous outcomes or a logistic model for categorical outcomes). Both primary patient outcome variables (PROMIS Global 10 and PAM) are continuous measures that are recorded at 7-days and at 90-days. The central parameter of interest is the difference between the mean within-subject differences (calculated for each subject) in the 2 intervention groups, compared to the usual care group. The statistical significance of the overall intervention effect will be obtained from the overall F statistic and pairwise comparison will be generated using Tukey’s least significant difference. For secondary dichotomous outcomes, a univariate logistic regression model will be used to generate odds ratios and 95% CIs comparing the two intervention groups to the usual care group.

If significant baseline differences are noted in important prognostic factors (e.g., age, sex, stroke type, stroke severity), we will adjust for these factors in multivariable analyses. Following the primary analysis, interaction effects between the intervention groups and key prognostic factors will be tested (including age, sex, stroke severity, type of caregiver, post-stroke function, duration between stroke onset and return home). Using sensitivity analyses we will assess the impact of using proxy respondents, multiple imputation (if used), and site-specific effects (using random effects models). Finally, multilevel modeling will be used to examine both between- and within-dyad differences [65].

## Methods: Monitoring

### Data monitoring

No interim statistical analyses are planned. Case accrual will continue until the target goal of 315 consented stroke patients is reached or until the end of the recruitment period (June 2017). After case recruitment begins in January 2016, the study team will actively monitor case enrollment at each of the study sites and will suggest modifications accordingly to ensure that enrollment targets are met. Similarly we will actively monitor the completion rates of other key processes including home visits, access and utilization of the MISTT website, completion of 7-day and 90-day outcome interviews, and intervention discontinuation. Given that safety outcomes in this study are limited (see below) the study will not utilize a Data Safety and Monitoring Committee. Instead information on case accrual, safety outcomes and other study process measures will be shared at regular meetings of a Patient-Professional Advisory Board. No external monitoring of the trial procedures or data collection processes will occur.

### Monitoring harms

There are two study-related potential risks to participants: 1) loss of confidentiality, and 2) increased stress as a result of the social work intervention addressing unmet needs and other biopsychosocial stressors. The protocol includes extensive protections to ensure confidentiality and mitigate the risk of loss of confidentiality. These include the use of study identification numbers (rather than names), secure password protected REDCap data systems, and storage of all hard copy PHI data in secure locations.

If, during the trial, a serious non-medical situation arises that suggests the patient is in crisis, the SWSCM will refer the patient to local crisis support services and ensure that the patient is connected to the appropriate services. All study personnel are instructed on the appropriate use of 9–1-1 services should an emergent medical situation arise during any of the patient or caregiver interactions. If other serious but non-emergent medical problems arise (for example, ran out of medications, evidence of severe depression score, or suicidal ideation) the SWSCM will make a recommendation for follow-up with appropriate services such as the patient’s primary care provider.

### Ethics and dissemination

All procedures conducted during this trial are carried out in compliance with federal and institutional ethical standards. All procedures and consent forms were approved by the Institutional Review Boards at the main study site (Michigan State University) as well as at the participating hospitals. Dissemination plans include the usual process of peer-review publications and presentations at scientific conferences. However, our dissemination activities will also engage local and regional stakeholders including hospitals, community-based care providers, public health agencies, and non-governmental community health agencies.

## Discussion

Recent efforts to re-design the medical care delivery system in the U.S. are focusing on the entire “episode of care”, [66] which will demand improvements in care transitions for stroke patients who return to their home setting [6, 24]. The MISTT study integrates core social work principles with health information technology and the Chronic Care Model (CCM) [67, 68]. The goal of these interventions is to actively engage stroke patients and their caregivers in the complex decision making and self-management required after returning home following the hospital [67, 69, 70]. The study intervenes on four components of the Chronic Care Model (CCM) – self-management support, community resources, delivery system redesign, and clinical information systems [67, 68]. As a guiding framework, the CCM aligns with elements identified as necessary for effective care transitions for patients with complex care needs, such as improving informational resources and providing better coordination of care across multiple settings [33, 71, 72]. Moreover, the core principles and roles of social workers are also congruent with the goals of the CCM and with the needs of patients during the transitional care period.

Case management programs utilizing social workers have shown positive outcomes for older adults transitioning from hospital to home [73–76]. These programs have many similarities with nurse-led case manager transitional models, [32, 33] including an emphasis on home-based assessments, follow-up medical appointments, patient and caregiver education, and accessing community resources. However, while nurse case manager programs focus primarily on health conditions, medication, and lifestyle changes, social workers add value by expanding patient and caregiver support for psychosocial issues, including tangible and emotional support [71, 77]. In addition to providing short-term counseling for emotional issues, social work case managers address barriers to accessing follow-up medical care and social services. This includes coordinating care across multiple health systems; problem-solving to address needs such as transportation and finances; education; monitoring progress; and advocating for patients. This is particularly important for patients with complex medical and psychosocial needs, many of which do not become apparent until after discharge [73, 76, 77]. Practical implementation of case management programs designed to improve the transition experience of stroke patients and caregivers requires an understanding of many issues including costs. The detailed documentation of the intensity of case management services (i.e., their type, frequency, and duration) provided in the interaction logs by the SWCM will enable the project to estimate program costs.

In summary, while some evidence for the positive effects of self-management programs to aid the recovery of stroke patients does exist, [78] currently no evidence-based programs are implemented as a standard of care to address the psychosocial and health-related challenges that stroke patients and their caregivers face during the transition period. The MISTT study will add to the evidence base by testing the efficacy of home based case management programs led by social workers, and by testing the value of online health information and resources to make a meaningful difference in the lives of stroke patients and their caregivers.

## Abbreviations

BCOS: Bakas Caregiving Outcomes Scale
MISTT: Michigan Stroke Transitions Trial
mRS: modified Rankin score
NeuroQoL: Quality of Life in Neurological Disorders
NIH: National Institutes of Health
OCBS: Oberst Caregiver Burden Scale
PCORI: Patient-Centered Outcomes Research Institute
PROMIS: Patient-Reported Outcomes Measurement Information System
SWSCM: Social worker stroke case management

## Footnotes

1
www.google.com/analytics

2
www.projectredcap.com
